# Michigan State Board of Health

**Published:** 1881-12

**Authors:** 


### ARTICLE VII.

### MICHIGAN STATE BOARD OF HEALTH.

The regular quarterly meeting of this Board was held Oct. 11, 1881. An interesting feature was a report by the Secretary relative to work of other State Boards of Health. The Secretary of the Michigan Board desires to continue to receive information from other Boards, by which these reports may be made quarterly.

A report relative to work of local boards of health showed increased activity on the part of local health authorities in the way of isolating those affected with communicable diseases, and enforcing the law requiring from householders and physicians notices of such diseases. In one city a physician had been fined $100 for not reporting cases of diphtheria.

The revised document on the restriction and prevention of scarlet fever was adopted, and ordered published in English, Dutch and German. The consideration of this document involved a discussion of the question of recommending health officers to verify diagnoses of reported cases of diseases dangerous to the public health.

A circular giving general rules for the prevention of diphtheria, scarlet fever and small-pox was adopted. Forms were adopted for annual reports by health officers and clerks of local boards of health, and by regular correspondents of the Board.

Dr. Avery, of Greenville, was requested to visit the overflowed district along the Maple river, in Gratiot county, and report to the Board.

Dr. Lyster, of Detroit, read a paper on syphilis in its relations to the public health. It dealt with the facts of the frequent communication of the contagium of syphilis, by direct and by indirect means, to innocent persons; also with the serious effects on individuals, and on the offspring of marriages, where one of the parents is thus blighted. He believed much might be done toward preventing this loathsome disease by wise legislation which shall restrict syphilis, and especially by collecting and disseminating among young men and other people facts relating to the nature and dangers of this disease.

Dr. Kellogg read a paper on the Relations of Preventable Sickness to Taxation, showing, by the reports of the boards of correction and charities, the abstracts of reports of county superintendents of the poor, the abstracts of statistical information relating to the insane, and the deaf, dumb and blind, and the vital statistics report, that more than 3,000 persons in Michigan are annually dependent on the State for support to a greater or less extent, in consequence of diseases preventable by the adoption of proper sanitary measures. The cost to the people of the State for the support of these persons is over $40,000 annually, a portion of which is paid by every tax-payer. This is but a small part of the actual loss to the State. The number of deaths from preventable sickness in 1880 (*estimated* from returns by supervisors and assessors), was 4,585. Placing the value to the State of each human being at the low estimate of $1,000, the aggregate loss by deaths from preventable sickness is over $4,500,000. But to this must be added a further loss from sickness which did not terminate fatally. The statistics of the benefit societies of England show that for every person who dies three persons (on the average) are sick, throughout the year. This indicates a total annual loss of time from preventable illness on the part of more than 9,000 persons, to which should be added the expense of living, etc., certainly more than $1,000,000. This gives about

$5,666,000 as the total loss to this State from diseases generally conceded to be preventable. These figures are regarded as much too small, because of the few diseases included in this estimate as preventable (though it is generally conceded by sanitarians that at least nine-tenths of all ailments may readily be prevented), and because only sickness and deaths directly traceable to preventable causes have been included, while a large amount of sickness and many deaths are indirectly due to these causes. It is probable that preventable sickness might justly be charged with an expense to the State of not less than ten million dollars. Estimating the loss in the other States in the same ratio to the population, the aggregate loss to the whole United States is not less than three hundred million dollars annually, an amount which would pay the national debt in six years.

Mr. Parker, of Flint, presented a report of the public health section of the American Social Science Association at Saratoga.

The committee on sanitary survey of the State was requested to prepare schedules for the sanitary survey of cities, villages and townships.

Mr. Parker reported a proposed bill authorizing all boards of education to exclude from school persons infected with diphtheria, scarlet fever or small-pox, or living at houses where any person is infected with one of these diseases.

The Secretary was directed to prepare and issue a weekly bulletin of sickness in Michigan for such papers and medical journals as will publish it.

Dr. Baker was authorized to procure the services of an architect in the preparation of a circular on hospitals for communicable diseases.

Dr. Kellogg reported on the subject of criminal abortion. He and Dr. Hazlewood were requested to prepare a circular designed to collect facts on this subject.

